# Inhibition of Host Cell Lysosome Spreading by Trypanosoma cruzi Metacyclic Stage-Specific Surface Molecule gp90 Downregulates Parasite Invasion

**DOI:** 10.1128/IAI.00302-17

**Published:** 2017-08-18

**Authors:** João Paulo Ferreira Rodrigues, Guilherme Hideki Takahashi Sant'ana, Maria Aparecida Juliano, Nobuko Yoshida

**Affiliations:** a Departamento de Microbiologia, Imunologia e Parasitologia, Escola Paulista de Medicina, Universidade Federal de São Paulo, São Paulo, SP, Brazil; b Departamento de Biofísica, Escola Paulista de Medicina, Universidade Federal de São Paulo, São Paulo, SP, Brazil; University of South Florida

**Keywords:** Trypanosoma cruzi, host cell invasion

## Abstract

Successful infection by Trypanosoma cruzi, the agent of Chagas' disease, is critically dependent on host cell invasion by metacyclic trypomastigote (MT) forms. Two main metacyclic stage-specific surface molecules, gp82 and gp90, play determinant roles in target cell invasion *in vitro* and in oral T. cruzi infection in mice. The structure and properties of gp82, which is highly conserved among T. cruzi strains, are well known. Information on gp90 is still rather sparse. Here, we attempted to fill that gap. gp90, purified from poorly invasive G strain MT and expressing gp90 at high levels, inhibited HeLa cell lysosome spreading and the gp82-mediated internalization of a highly invasive CL strain MT expressing low levels of a diverse gp90 molecule. A recombinant protein containing the conserved C-terminal domain of gp90 exhibited the same properties as the native G strain gp90: it counteracted the host cell lysosome spreading induced by recombinant gp82 and exhibited an inhibitory effect on HeLa cell invasion by CL strain MT. Assays to identify the gp90 sequence associated with the property of downregulating MT invasion, using synthetic peptides spanning the gp90 C-terminal domain, revealed the sequence GVLYTADKEW. These data, plus the findings that lysosome spreading was induced upon HeLa cell interaction with CL strain MT, but not with G strain MT, and that in mixed infection CL strain MT internalization was inhibited by G strain MT, suggest that the inhibition of target cell lysosome spreading is the mechanism by which the gp90 molecule exerts its downregulatory role.

## INTRODUCTION

Although the number of people infected with Trypanosoma cruzi, the protozoan parasite that causes Chagas' disease, has declined over the years, there are still problems that challenge the control of the disease. T. cruzi infection is no longer restricted to poor rural populations in Latin America but is becoming a worldwide health problem due to population migration to countries where the disease is not endemic, where transmission is associated mainly with blood transfusion (1, 2). Another problem of considerable public health concern, particularly in the last 10 years, has been the frequent outbreaks of acute Chagas' disease by oral transmission in Brazil, Colombia, and Venezuela (3). The parasite form implicated in oral infection is the metacyclic trypomastigote (MT) from triatomine insects (4). Two main metacyclic stage-specific surface molecules, gp82 and gp90, have been shown to play determinant roles in MT invasion of host cells *in vitro* as well as in oral T. cruzi infection in mice (5–8). gp82 and gp90 are members of the gp85/*trans*-sialidase superfamily (9, 10), which bind to host cells in a receptor-mediated manner and induce opposite effects. In contrast to gp82, which mediates MT internalization by inducing Ca^2+^ signal in host cells, leading to actin cytoskeleton disruption (11), lysosome spreading, and exocytosis (12, 13), events that contribute to parasitophorous vacuole formation (14, 15), gp90 is devoid of such properties (16, 17) and functions as a negative regulator of MT invasion (18).

Studies with MT of T. cruzi strains CL and G, which belong to divergent genetic groups (19) and differ widely in their ability to invade host cells *in vitro* and to infect mice (20, 21), have shown that the gp82 expressed in these strains share 97.9% overall sequence identity and 100% identity regarding the central domain containing the host cell binding sites (21). Although expressing gp82 at similar levels (16, 22), the ability of G strain MT to enter different mammalian cell types is much lower than that of CL strain MT, presumably because the gp90-mediated interaction with target cells prevails over that mediated by gp82 (21). Differently from gp82, there appears to be a higher diversity in gp90 proteins expressed in different strains. The gp90 recognized by monoclonal antibody 1G7 (MAb 1G7) is expressed in G and other T. cruzi strains of genetic group TcI, either from the wild transmission cycle or from chagasic patients (16, 22), whereas the gp90 reactive with MAb 5E7, but not with MAb 1G7, is expressed in CL and other T. cruzi strains from the domestic transmission cycle (6, 7). Regardless of the gp90 isoform, there is a close association between the high gp90 levels and reduced capacity to enter host cells (6, 7, 22). Unknown is the degree of similarity between the diverse gp90 molecules or whether there is a common motif recognized by target cells. To address these questions, we analyzed in this study the structure and properties of gp90 that are relevant for interaction with host cells and MT invasion.

## RESULTS

### Metacyclic forms of highly invasive T. cruzi strain CL induce host cell lysosome spreading.

**FIG 1:**
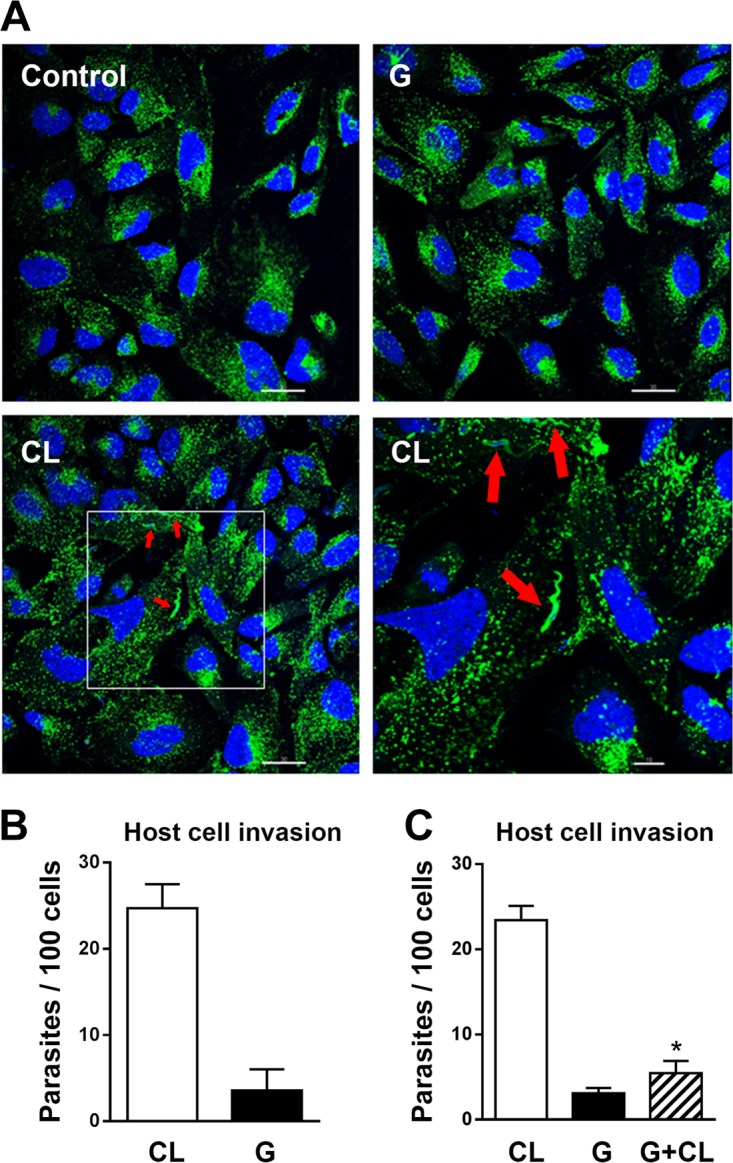
Host cell lysosome spreading is induced by MT of highly invasive T. cruzi CL strain but not by poorly invasive G strain. (A) HeLa cells were incubated for 1 h with MT of CL or G strain, along with control cells, and then processed for confocal fluorescence analysis using anti-LAMP2 antibody, Alexa Fluor 488-conjugated anti-mouse IgG (green), and DAPI (blue) for DNA, with a 63× objective. Scale bar, 30 μm. Note the lysosome scattering induced by CL strain MT and parasites associated with lysosome markers (red arrows), whereas lysosomes remained concentrated at the perinuclear area upon interaction with G strain MT. The framed region indicates the position of the magnified field shown in the lower right image. Scale bar, 10 μm. (B) HeLa cells were incubated for 1 h with MT of CL or G strain and then processed for intracellular parasite counting. Values are the means ± standard deviations (SD) from four independent assays performed in duplicate. (C) HeLa cells were incubated for 1 h with MT of CL or G strain alone or with both strains. Values are the means ± SD from three independent assays performed in duplicate. CL strain internalization was significantly inhibited in mixed infection (*, *P* = 0.0001).

Efficient cell invasion by MT has been associated with the parasite's ability to induce lysosome spreading (12, 13). By confocal immunofluorescence analysis, using anti-LAMP2 antibodies, we have observed that HeLa cell lysosomes are mobilized from the perinuclear area to the cell periphery upon 30 min of incubation with CL strain MT, whereas the interaction with G strain MT does not substantially change the lysosome distribution (Fig. 1A). This result is compatible with the differential invasion capacity of the two strains. Invariably, the number of internalized CL strain MT (multiplicity of infection [MOI] of 10) upon 1 h of incubation with HeLa cells has been found to be more than 6-fold higher than that of G strain at an MOI of 20 (Fig. 1B), and it was always in association with lysosome markers (Fig. 1A). As the low invasion capacity of G strain MT is attributed to its property in downregulating the host cell signaling pathways that lead to lysosome spreading, we asked whether G strain MT could exert an inhibitory effect on CL strain MT internalization in a mixed infection. HeLa cells were incubated for 15 min with G strain MT (MOI of 20), and then CL strain parasites (MOI of 10) were added and the incubation proceeded for 1 h. Compared to the control single infection, CL strain internalization was significantly inhibited in mixed infection, reaching values similar to those of invasion by G strain (Fig. 1C).

### CL and G strain MT express distinct gp90 molecules that share conserved structural features.

**FIG 2:**
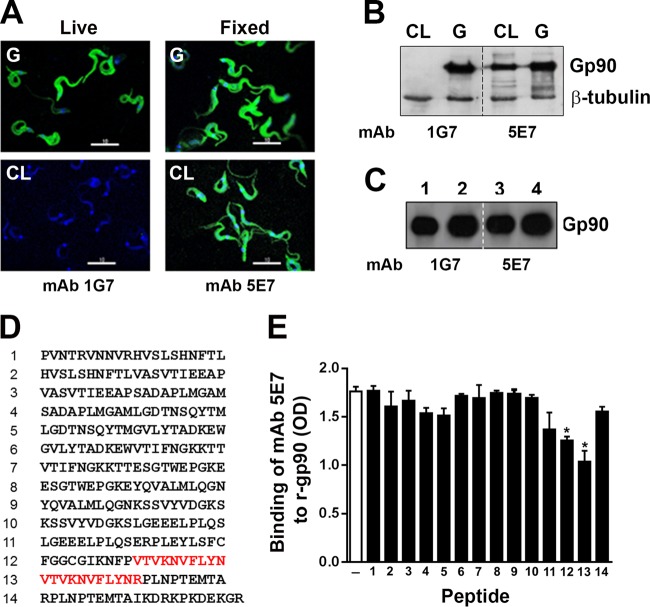
CL and G strain MT express distinct gp90 molecules that share conserved structural features. (A) Live parasites were incubated for 1 h with MAb 1G7. Following fixation and reaction with Alexa Fluor 488-conjugated anti-IgG (green) and DAPI (blue), the parasites were visualized with a confocal microscope with a 100× objective. Alternatively, parasites were fixed before reaction with MAb 5E7 and then processed as described above. Scale bar, 10 μm. Note the lack of reaction of CL strain MT with MAb 1G7. (B) Western blots of detergent-soluble MT extracts revealed by the indicated monoclonal antibodies. Note the lower intensity of the CL strain gp90 band recognized by MAb 5E7. (C) Protein A/G magnetic beads, cross-linked to MAb 1G7, were incubated for 1 h with G strain MT lysates. After washings, the proteins bound to beads were eluted and subjected to SDS-PAGE and Western blotting, along with unbound proteins. Eluates from MAb 1G7 beads (lanes 1 and 3) and unbound proteins (lanes 2 and 4) were revealed with MAb 1G7 or MAb 5E7. (D) Sequences of peptides spanning the C-terminal domain of g90. (E) Microtiter plates coated with a recombinant protein containing the C-terminal portion of gp90 (r-gp90C) were incubated with MAb 5E7 in the absence or in the presence of 100 μg/ml individual peptides shown in panel D. Binding of MAb 5E7 was measured by ELISA. Values are the means ± SD from triplicates. The sequences shared by peptides 12 and 13, which inhibited antibody binding, are highlighted in red in panel D. *, *P* = 0.0005.

The surface molecule gp90 is thought to be primarily responsible for the downregulatory effect of G strain MT on target cells (21). gp90 is also expressed in CL strain MT, but it differs from that expressed in G strain for lack of recognition by MAb 1G7 (16). In contrast to a strong reaction detectable by immunofluorescence analysis, upon 1 h of incubation of live G strain MT with MAb 1G7 on ice, followed by fixation with 4% paraformaldehyde (Fig. 2A) or in Western blots of detergent MT extracts (Fig. 2B), reaction with CL strain was consistently negative. Common to both strains is the reactivity with MAb 5E7, which does not recognize live MT, only fixed parasites (Fig. 2A), indicating that the native gp90 epitope for MAb 5E7 is cryptic. Reaction of fixed MT with unrelated MAb 1D9 directed to an amastigote-specific epitope was negative (see Fig. S1 in the supplemental material). In Western blotting, the MAb 5E7-reactive gp90 is detected as a band of higher intensity in G strain than in CL strain, and this is not due to unequal sample loading, as indicated by similar intensities of the band corresponding to β-tubulin, which was used as a loading control (Fig. 2B). gp90 is also released in large amounts by G strain MT and may contribute to further downregulating parasite invasion (17). We compared the MAb 5E7-reactive gp90 released into medium by CL and G strain MT after 15, 30, and 60 min of incubation by analyzing the supernatant collected following centrifugation of parasites. G strain gp90 was released in large amounts as early as 15 min, as opposed to CL strain gp90, which was released in very small amounts (Fig. S2). The question of whether the MAb 1G7-reactive gp90 contains the epitope for MAb 5E7 was also examined. Protein A/G magnetic beads cross-linked with MAb 1G7 were incubated for 1 h with detergent-soluble lysates of G strain MT. After washings, proteins that bound to beads were eluted and equal amounts were loaded into an SDS-PAGE gel (Fig. 2C, lanes 1 and 3), along with unbound MT protein samples (lanes 2 and 4). After electrophoresis and transfer to the nitrocellulose membrane, the portion of the membrane comprising lanes 1 and 2 was incubated with MAb 1G7, whereas the portion comprising lanes 3 and 4 was incubated with MAb 5E7. A 90-kDa band of high intensity was detected in the eluate from MAb 1G7 beads by MAb 1G7 or 5E7 (lanes 1 and 3), indicating that the MAb 1G7-reactive gp90 of G strain MT contains the epitope for MAb 5E7. The MAb 1G7 failed to recognize a recombinant protein containing the C-terminal portion of gp90 (r-gp90C) (Fig. S3). To identify the MAb 5E7-reactive epitope, which is known to reside in the C-terminal domain of p90 (10), enzyme-linked immunosorbent assays (ELISAs) of inhibition of antibody binding were performed, using synthetic 20-mer peptides, with overlapping of 10 amino acid residues spanning the referred region (Fig. 2D). Microtiter plates coated with r-gp90C were incubated with MAb 5E7 in the absence or in the presence of individual peptides. Binding of MAb 5E7 to r-gp90C was significantly inhibited by peptides 12 and 13 (Fig. 2E). Both peptides contain the VTVKNVFLYN motif, which is highly conserved in gp85/*trans*-sialidase superfamily (23). In the structure model of gp82, this motif was shown to be mostly buried because of its content of amino acids that are barely accessible to solvent (24). This is in accord with the lack of reaction of MAb 5E7 with the native gp90.

### gp90 molecule from G strain MT inhibits host cell lysosome spreading and inhibits CL strain MT internalization.

**FIG 3:**
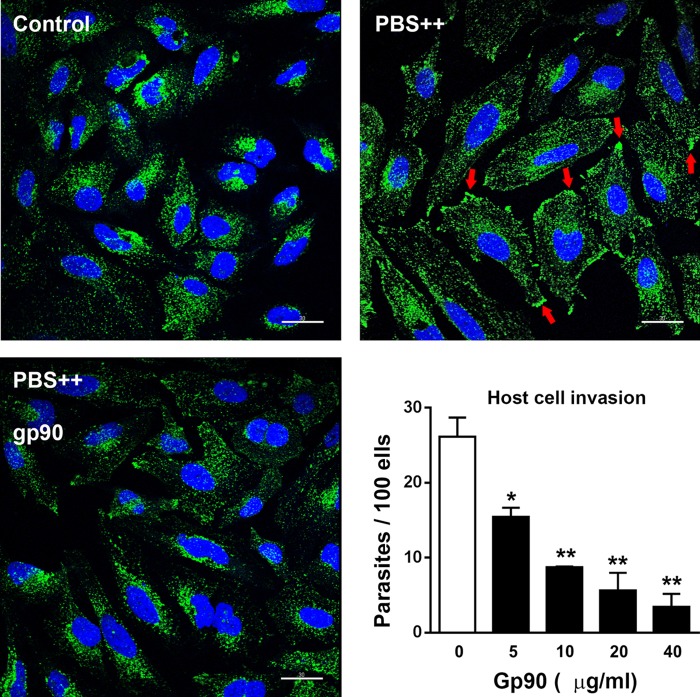
Host cell lysosome spreading and CL strain MT invasion are inhibited by gp90 molecule from G strain MT. HeLa cells were incubated for 30 min in complete medium (control) or in PBS^++^, in the absence or in the presence of 20 μg/ml gp90 purified from G strain MT, and processed for confocal fluorescence analysis using anti-LAMP2 antibodies as described for Fig. 1A. Scale bar, 30 μm. Note the lysosome spreading upon incubation in PBS^++^, with accumulation of lysosome markers at the cell borders (red arrows), and the inhibitory effect of gp90. The graph shows the rate of CL strain MT invasion upon 1 h of incubation of parasites with HeLa cells in the absence or in the presence of gp90 purified from G strain MT at the indicated concentrations. Values are the means ± SD from three independent assays performed in duplicate. gp90 significantly inhibited MT internalization at all concentrations (*, *P* < 0.005; **, *P* ≤ 0.0005).

To determine the effect of gp90 purified from G strain MT on target cell lysosome spreading and invasion by CL strain MT, HeLa cells were incubated for 30 min in complete medium or in phosphate-buffered saline containing Ca^2+^ and Mg^2+^ (PBS^++^) in the absence or in the presence of 20 μg/ml gp90 and processed for immunofluorescence using anti-LAMP2 antibodies. PBS^++^ was used because a short-term incubation of HeLa cells in this starvation medium results in extensive lysosome mobilization and increases the susceptibility to MT invasion (12, 25). gp90 counteracted the lysosome-spreading effect of PBS^++^ by retaining the lysosomes predominantly in the perinuclear area (Fig. 3). In the absence of gp90, lysosomes were scattered all over the cell, with a tendency to accumulate at the cell borders (Fig. 3), which are the preferential sites for T. cruzi invasion (26). To check the effect of gp90 on CL strain MT internalization, HeLa cells were treated for 15 min with gp90 at various concentrations, and then, without washing, the parasites were added. After 1 h of incubation, the cells were processed for intracellular parasite counting. gp90 decreased the cell susceptibility to MT invasion in a dose-dependent manner (Fig. 3, graph). At 40 μg/ml, gp90 was capable of reducing CL strain invasion to levels comparable to that of G strain (Fig. 3, graph).

### Lysosome spreading induced by gp82 and CL strain MT invasion are inhibited by r-gp90C.

**FIG 4:**
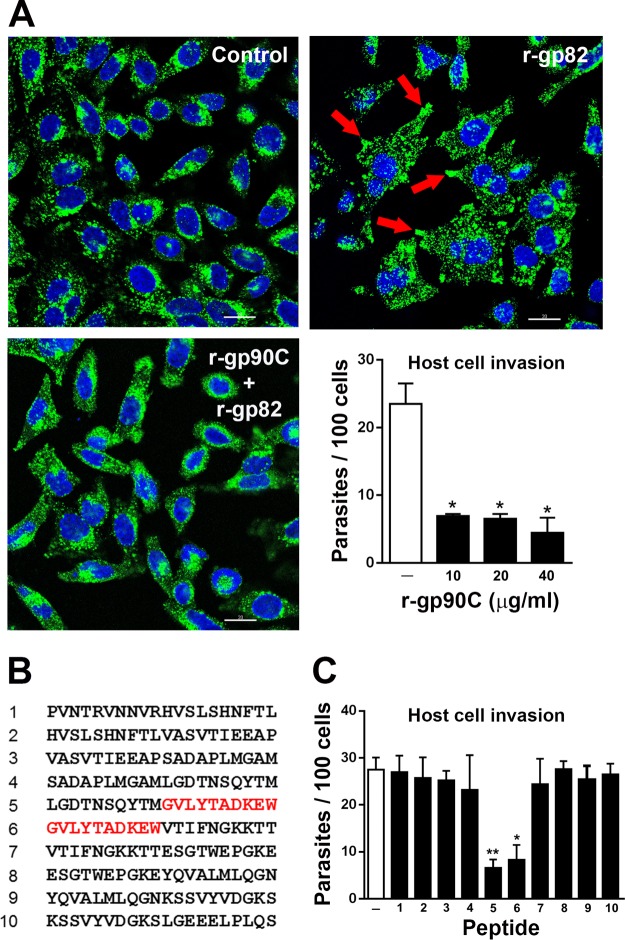
Recombinant protein r-gp90C counteracts host cell lysosome spreading induced by recombinant protein r-gp82 and CL strain MT internalization. (A) HeLa cells were incubated for 30 min with r-gp82 alone or mixed with r-gp90C and processed for immunofluorescence as described for Fig. 1A. Note the spreading of lysosomes induced by r-gp82, with accumulation at the cell edges (red arrows) and the lysosome retention at the perinuclear region in the presence of r-gp90C. The graph shows the rate of CL strain MT invasion upon a 1-h incubation of parasites with HeLa cells in the absence or in the presence of r-gp90C at the indicated concentrations. Values are the means ± SD from three independent assays performed in duplicate. MT internalization was significantly inhibited at all concentrations (*, *P* < 0.001). (B) Sequences of peptides comprising the gp90 C-terminal domain. (C) HeLa cells incubated for 1 h with CL strain MT in the absence or in the presence of 50 μg/ml individual peptides shown in panel B and processed for intracellular parasite counting. Values are the means ± SD from three assays performed in duplicate. The sequences shared by peptides 12 and 13, which inhibited MT internalization, are highlighted in red in panel B. *, *P* < 0.005; **, *P* < 0.0005.

We examined whether r-gp90C could counteract the activity of gp82 in inducing host cell lysosome spreading and in mediating CL strain MT invasion. HeLa cells were incubated for 30 min either with the recombinant protein containing the full-length gp82 sequence (r-gp82) alone or mixed with r-gp90C and then were processed for immunofluorescence to visualize lysosome distribution. Lysosome spreading was observed in cells incubated with r-gp82 alone, whereas the simultaneous incubation with r-gp90C retained the lysosomes in the perinuclear area (Fig. 4A) in a manner similar to that of the native gp90 (Fig. 3). In cell invasion assays, r-gp90C strongly inhibited CL strain MT internalization (Fig. 4A, graph), exhibiting an effect similar to that of the native gp90 (Fig. 3, graph). To identify the gp90 sequence associated with the property of downregulating MT invasion, we used synthetic peptides spanning the gp90 C-terminal domain, excluding the peptides corresponding to nonexposed sequences (Fig. 4B). HeLa cells were incubated for 1 h with CL strain MT in the absence or in the presence of individual peptides, at 50 μg/ml, and processed for intracellular parasite counting. Peptides 5 and 6, sharing the sequence GVLYTADKEW, significantly inhibited CL strain MT invasion (Fig. 4C).

### G strain MT internalization is inhibited by r-gp82 and r-gp90C, which have opposite effects on LAMP2 expression.

**FIG 5:**
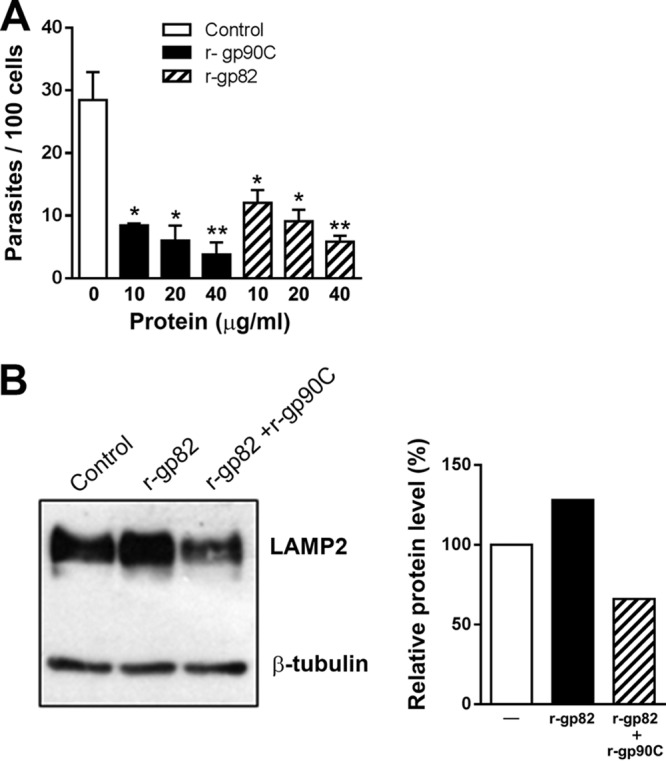
G strain MT internalization is inhibited by r-gp82 and r-gp90C, which have opposite effects on LAMP2 expression. (A) HeLa cells were incubated for 1 h with G strain MT in the absence or in the presence of r-gp90C or r-gp82, at various concentrations, and then processed for intracellular parasite counting. Values are the means ± SD from three independent assays performed in duplicate. MT invasion was significantly inhibited by r-gp90C and r-gp82 at all concentrations (*, *P* < 0.005; **, *P* < 0.001). (B) HeLa cells were incubated for 30 min in the absence or in the presence of r-gp82 alone or r-gp82 plus r-gp90C, and the detergent extract was analyzed by Western blotting using anti-LAMP-2 and anti-β-tubulin antibodies. The band intensities were measured with ImageJ software. LAMP2 intensity was normalized to the untreated control value, which was set equal to 100%. A representative result out of three is shown.

Experiments were performed to compare the effect of exogenous gp82 and gp90 on G strain MT invasion using the recombinant proteins r-gp82 and r-gp90C. HeLa cells were incubated for 1 h in PBS^++^ in the absence or in the presence of various protein concentrations. Both r-gp82 and r-gp90C significantly inhibited MT internalization in a dose-dependent manner (Fig. 5A). The number of intracellular parasites was 5-fold and 7.5-fold lower in the presence of 40 μg/ml r-gp82 and r-gp90C, respectively. As r-gp82 induces lysosome spreading (Fig. 4A), which is associated with an increased expression of the major lysosome membrane protein LAMP (13), we examined the ability of r-gp90C to counteract this effect. HeLa cells were incubated for 30 min in the absence or in the presence of r-gp82 alone or r-gp82 plus r-gp90C. The detergent extract then was analyzed by Western blotting using anti-LAMP2 antibody and anti-β-tubulin antibody as a loading control. In cells treated with r-gp82, the intensity of LAMP2 increased, but this effect was reversed by r-gp90C (Fig. 5B). Analysis by densitometry showed a LAMP2 level of about 130% and 65% in cells treated with r-gp82 and r-gp82 plus r-gp90C, respectively, relative to 100% of the untreated control (Fig. 5B). Similar results were obtained in repeated experiments. Taken together, the data from experiments using recombinant proteins indicate that gp82 and gp90 differentially interact with target cells, exerting opposite effects.

## DISCUSSION

Our findings indicate that the T. cruzi MT-specific surface molecule gp90 exerts a downregulatory role in parasite internalization mainly through its ability to inhibit host cell lysosome spreading.

Metacyclic forms of CL strain efficiently invade host cells by engaging the surface molecule gp82, which induces lysosome spreading, unimpaired by gp90 molecules, which are expressed and released into medium at low levels. The reduced capacity of G strain MT to enter host cells would be due mainly to large gp90 amounts, not only present on the parasite surface but also released into medium, which strongly inhibit lysosome spreading. Support for these assertions is provided by a number of findings. Under conditions that mimicked G strain MT-host cell interaction, such as the presence of G strain gp90 in the medium, CL strain MT internalization was significantly diminished. We presume that the cell invasion capacity of CL strain MT would be similar to that of G strain MT if they expressed and released gp90 at high levels. The fact that diverse gp90 molecules are expressed in CL and G strain MT does not invalidate this assumption, because the gp90 C-terminal domain is highly conserved (27) and contains the sites relevant for interaction with target cells. As a matter of fact, the recombinant protein r-gp90C was capable of inhibiting target cell lysosome spreading and invasion by CL strain MT in the same manner as the native gp90. Within the gp90 C-terminal domain, the sequence GVLYTADKEW may play a key role in mediating gp90 interaction with target cells and downregulating MT invasion. In gp82, whose C-terminal domain shares 44.5% sequence identity with the corresponding domain in gp90, the GVLYTADKEW motif is substituted for GLSYGTDGTW. The present findings on gp90 and previous data on gp82 reinforce the notion that gp90 and gp82 are recognized by distinct host cell receptors that relay different signals that render the cells more susceptible or resistant to MT invasion.

gp90 plays a critical role in the establishment of T. cruzi infection by the oral route and the courses of infection may vary, depending on which isoform is expressed in a given parasite strain (6, 7). When administered orally into mice, MT of different T. cruzi strains reach the stomach and invade the gastric epithelial cells (28). Metacyclic forms of the CL strain efficiently invade the gastric epithelium, where the parasites replicate and subsequently trypomastigotes are detectable in the bloodstream, whereas T. cruzi strains with surface profiles similar to that of the CL strain, except for the expression of gp90 at high levels, exhibited quite distinct invasion properties. Strains expressing gp90 susceptible to degradation by pepsin at acidic pH entered gastric epithelial cells at high rates, whereas the strain expressing gp90 resistant to peptic digestion was poorly internalized (6, 7). On the other hand, a characteristic common to G strain and several other strains belonging to the genetic group TcI is the expression at high levels of a pepsin-resistant r-gp90 and the reduced capacity to invade the gastric epithelium (7, 22). Differently from the variability of gp90, the gp82 molecule of different strains is resistant to peptic digestion (6, 7, 22), which is compatible with the highly conserved sequence of gp82 expressed in diverse genetic groups (21). Taken together, the data on gp90 molecules suggest that they play a decisive role in modulating T. cruzi infection.

## MATERIALS AND METHODS

### Parasites and host cell invasion assay.

T. cruzi strains CL and G were maintained either in mice or in liver infusion tryptose (LIT) medium. G strain metacyclic forms were obtained from LIT cultures at the stationary growth phase. The CL strain was grown for one passage in Grace's medium (Life Technologies) to stimulate differentiation to MT. Metacyclic forms purified by passage through DEAE-cellulose columns, as described previously (29), were used for cell invasion assays with HeLa cells, the human carcinoma-derived epithelial cells grown at 37°C in Dulbecco's minimum essential medium (DMEM), supplemented with 10% fetal calf serum (FCS), streptomycin (100 μg/ml), and penicillin (100 U/ml) in a humidified 5% CO_2_ atmosphere. Parasites were seeded onto 24-well plates containing 13-mm-diameter round glass coverslips coated with 1.4 × 10^5^ HeLa cells (MOI of 10 for CL strain and MOI of 20 for G strain). After 1 h of incubation, the duplicate coverslips were fixed in Bouin solution, stained with Giemsa, and sequentially dehydrated in acetone, a graded series of acetone-xylol (9:1, 7:3, 3:7), and xylol. The number of intracellular parasites was counted in a total of 250 cells.

### Preparation and purification of native and recombinant MT proteins.

Native gp90 was purified from detergent-soluble extract of G strain MT by affinity chromatography on immobilized MAb 1G7, as described previously (30). The recombinant protein containing the C-terminal domain of T. cruzi gp90 (GenBank accession number L11287) and the recombinant protein containing the full-length gp82 sequence (GenBank accession number L14824), both in fusion with glutathione *S*-transferase (GST), were produced in Escherichia coli as described previously (10, 11). All steps for the purification of the recombinant proteins followed a previously described procedure (11), and the purified protein was analyzed by Coomassie blue staining of SDS-PAGE gels and by immunoblotting using monoclonal antibody directed to gp90 or gp82.

### Microscopic visualization of fluorescent parasites.

Live metacyclic forms were incubated for 1 h on ice with anti-gp90 MAb, washed, and fixed with 4% paraformaldehyde, followed by washings and treatment with 50 mM NH_4_Cl in PBS for 30 min. After washings in PBS, the parasites were placed onto glass slides and dried. Afterwards, the parasites were incubated for 1 h with Alexa Fluor 488-conjugated anti-mouse IgG (Cell Signaling) and diluted 1:500 in PGN (0.15% gelatin in PBS containing 0.1% sodium azide) plus 10 μM DAPI (4′,6′1-diamino-2-phenylindole dihydrochloride) (Sigma) for visualization of kinetoplast and nucleus. In parallel, the parasites first were fixed with 4% paraformaldehyde in PBS, washed, and then placed onto glass slides and dried. After reaction with anti-gp90 MAb, the slides were processed as described above. Confocal images were acquired with a Leica TCS SP8 laser-scanning microscope (Leica, Germany) using a 100× oil immersion Plan Apochromat.

### Visualization of HeLa cell lysosomes by confocal microscopy.

Coverslips with adherent HeLa cells were incubated for 30 min at 37°C with parasites. After fixation with 4% paraformaldehyde in PBS for 20 min, treatment with 50 mM NH_4_Cl in PBS, and washes in PBS, the cells were incubated for 1 h at room temperature with mouse anti-human LAMP2 diluted 1:8 (vol/vol) in PGN containing 1% saponin (PGN-saponin). Following washes in PBS, the coverslips were incubated for 1 h with Alexa Fluor 488-conjugated anti-mouse IgG (Invitrogen) and diluted 1:500 in PGN-saponin containing 10 μg/ml DAPI, and the coverslips were mounted in ProLong Gold (Invitrogen). Confocal images were acquired in a confocal microscope as described above, using a 63× objective. The series of images obtained from confocal z-stacks were processed and analyzed using Leica LAS AF (Leica, Germany) and Imaris (Bitplane) software.

### Identification of gp90 epitopes for MAb 5E7.

Microtiter plates, coated with the recombinant protein containing the C-terminal domain of gp90 (1 μg/well), were incubated for 1 h with MAb 5E7 in the absence or in the presence of 100 μg/ml individual peptides spanning the referred domain (Fig. 2D) in PBS containing 2 mg/ml bovine serum albumin (PBS-BSA). After washings in PBS, the plates were incubated with peroxidase-conjugated anti-mouse IgG diluted in PBS-BSA. The final reaction was revealed by *o*-phenylenediamine and the absorbance at 492 nm read in an absorbance microplate reader.

### Immunoprecipitation and Western blotting.

Purified metacyclic forms were lysed with PBS containing 0.5% nonionic detergent Igepal CA630 (USB Corporation), and cleared lysates were used for immunoprecipitation. A/G magnetic beads (Pierce cross-link magnetic immunoprecipitation/coimmunoprecipitation [IP/co-IP] kit) cross-linked with MAb 1G7, according to the manufacturer's instructions (Thermo Scientific), were incubated for 1 h with parasite lysates at room temperature under constant agitation. Following washes with PBS containing 0.25% IGEPAL CA630, the bound proteins were eluted with the elution buffer, pH 2, and subjected to SDS-PAGE followed by Western blotting using MAb 1G7 or MAb 5E7. Western blot analysis for detection of LAMP2 protein was performed with detergent extract of HeLa cells incubated under different conditions.

### Preparation of parasite supernatant.

Metacyclic forms of parasites (10^8^) were incubated at 37°C for various times. After centrifugation, the pellet was discarded and the supernatant was collected. For Western blotting, 10 μl supernatant was loaded into the gel and the blots were revealed with MAb 5E7.

### Statistical analysis.

The Student *t* test, as implemented in GraphPad Prism software (version 6.01), was employed.

### Ethics statement.

All procedures conformed to Brazilian National Committee on Ethics Research (CONEP) guidelines, and the study was approved by the ethical committee for animal experimentation of the Universidade Federal de São Paulo (protocol no. CEUA9933271016).

## Supplementary Material

Supplemental material
