# Supplementary material for: Inhibition of Host Cell Lysosome Spreading by Trypanosoma cruzi Metacyclic Stage-Specific Surface Molecule gp90 Downregulates Parasite Invasion
Source: Infect Immun. 2017 Aug 18;85(9):e00302-17. doi: 10.1128/IAI.00302-17 (PMC5563561; doi:10.1128/IAI.00302-17)
Supplement: Supplemental material [file supp_85_9_e00302-17__index.html]

Inhibition of Host Cell Lysosome Spreading by Trypanosoma cruzi Metacyclic Stage-Specific Surface Molecule gp90 Downregulates Parasite Invasion — Supplemental material 

# Inhibition of Host Cell Lysosome Spreading by Trypanosoma cruzi Metacyclic Stage-Specific Surface Molecule gp90 Downregulates Parasite Invasion

## Supplemental material

- Supplemental file 1 -

  Fig. S1. Recognition of *T. cruzi* metacyclic forms by MAb 5E7 and lack of reaction with unrelated MAb 1D9. Fig. S2. Differential release of gp90 molecules into medium by MT of CL and G strains. Fig. S3. Lack of recognition of r-gp90C by MAb 1G7.

  PDF, 235K
