# Supplementary material for: Inhibition of Host Cell Lysosome Spreading by Trypanosoma cruzi Metacyclic Stage-Specific Surface Molecule gp90 Downregulates Parasite Invasion
Source: Infect Immun. 2017 Aug 18;85(9):e00302-17. doi: 10.1128/IAI.00302-17 (PMC5563561; doi:10.1128/IAI.00302-17)

Legend to Supplementary Figures

Supplementary Figure S1. Recognition of *T. cruzi* metacyclic forms by mAb 5E7 and lack of reaction with unrelated mAb 1D9. G strain metacyclic forms were fixed and then incubated for 1 h with mAb 5E7 or with an unrelated mAb 1D9 directed to an amastigote-specific epitope. After processing for immunofluorescence, the parasites were visualized in a confocal microscope. Scale bar = 10 µm. Note the lack of reaction of parasites with mAb 1D9.

Supplementary Figure S2. Differential release of gp90 molecules into medium by MT of CL and G strains. Parasites were incubated for 15, 30 and 60 min in culture medium. The Western blot of conditioned medium generated at 15, 30 and 60 min was probed with mAb 5E7. Note the high amounts of gp90 released by G strain.

Supplementary Figure S3. Lack of recognition of r-gp90C by mAb 1G7. Western blot containing the recombinant protein r-gp90C was probed with mAb 1G7 or mAb 5E7. Note that mAb 1G7 failed to react with r-gp90C.

Supplementary Figure S1

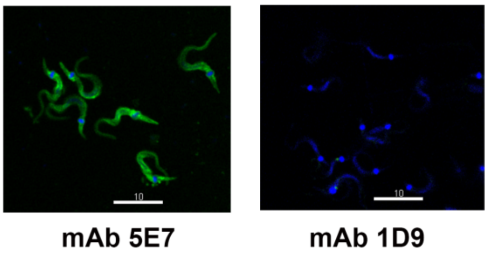

Supplementary Figure S2

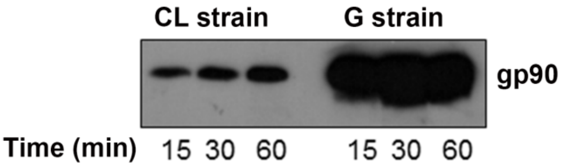

Supplementary Figure S3

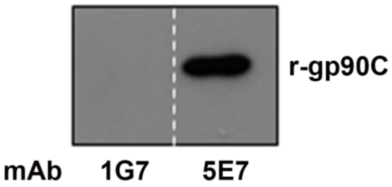

Supplement: Supplemental material [file IAI.00302-17_zii999092112s1.pdf]
